# A new reporter mouse cytomegalovirus reveals maintained immediate-early gene expression but poor virus replication in cycling liver sinusoidal endothelial cells

**DOI:** 10.1186/1743-422X-10-197

**Published:** 2013-06-17

**Authors:** Franziska Dag, Adrien Weingärtner, Milada Butueva, Ianina Conte, Julia Holzki, Tobias May, Barbara Adler, Dagmar Wirth, Luka Cicin-Sain

**Affiliations:** 1 Department of Vaccinology and Allied Microbiology, Helmholtz Centre for Infection Research, Inhoffenstr. 7, 38124 Braunschweig, Germany; 2 Research group ‘Model Systems for Infection and Immunity’, Helmholtz Centre for Infection Research, Braunschweig, Germany; 3 InScreenEx GmbH, Braunschweig, Germany; 4 Max von Pettenkofer Institute, Ludwig Maximillian University, Munich, Germany; 5 Institute for Virology, Medical School Hannover, Hannover, Germany

**Keywords:** Mouse cytomegalovirus, Major immediate early promoter, Conditionally immortalized cell line, Liver sinusoidal endothelial cells

## Abstract

**Background:**

The MCMV major immediate early promoter/enhancer (MIEP) is a bidirectional promoter that drives the expression of the three immediate early viral genes, namely ie1, ie2 and ie3. The regulation of their expression is intensively studied, but still incompletely understood.

**Methods:**

We constructed a reporter MCMV, (MCMV-MIEP^r^) expressing YFP and tdTomato under the control of the MIEP as proxies of ie1 and ie2, respectively. Moreover, we generated a liver sinusoidal endothelial cell line (LSEC-uniLT) where cycling is dependent on doxycycline. We used these novel tools to study the kinetics of MIEP-driven gene expression in the context of infection and at the single cell level by flow cytometry and by live imaging of proliferating and G_0_-arrested cells.

**Results:**

MCMV replicated to higher titers in G_0_-arrested LSEC, and cycling cells showed less cytopathic effect or YFP and tdTomato expression at 5 days post infection. In the first 24 h post infection, however, there was no difference in MIEP activity in cycling or G_0_-arrested cells, although we could observe different profiles of MIEP gene expression in different cell types, like LSECs, fibroblasts or macrophages. We monitored infected LSEC-uniLT in G_0_ by time lapse microscopy over five days and noticed that most cells survived infection for at least 96 h, arguing that quick lysis of infected cells could not account for the spread of the virus. Interestingly, we noticed a strong correlation between the ratio of median YFP and tdTomato expression and length of survival of infected cells.

**Conclusion:**

By means of our newly developed genetic tools, we showed that the expression pattern of MCMV IE1 and IE2 genes differs between macrophages, endothelial cells and fibroblasts. Substantial and cell-cycle independent differences in the ie1 and ie2 transcription could also be observed within individual cells of the same population, and marked ie2 gene expression was associated with longer survival of the infected cells.

## Background

Human Cytomegalovirus (HCMV) is an opportunistic pathogen belonging to the family of herpesviridae that infects the majority of the human population. It is the most frequent infectious cause of malformations and a major morbidity risk for immunosuppressed or immunodeficient patients. Like all cytomegaloviruses, HCMV is strictly species specific and is able to replicate only in human cells. Therefore, *in vivo* experiments with HCMV are difficult and rely on humanized mouse models. On the other hand, HCMV shares many similarities with the murine cytomegalovirus (MCMV) [1,2] and MCMV has been used as a model for HCMV in numerous studies.

Immediately upon infection, both the HCMV and the MCMV express viral genes controlled by the major immediate early promoter/enhancer (MIEP) at high levels [1,3], and their transcripts are detected as early as one hour post infection [4]. Deletion of the human IE1 and the murine ie1 genes affects the viral growth *in vitro* at low MOIs [5-7]. Although these proteins are not essential for viral replication, they are known to co-localize with nuclear domains 10 (ND10) and to disperse these complexes known for their antiviral activity [8-10]. Moreover, it was shown that MCMV ie1 plays a role in the transactivation of host ribonucloetide reductase and thymidylate synthase [11] genes. The alternatively spliced MCMV ie3, and its HCMV homologue IE2, are essential for viral replication and act as transactivators of viral early genes [12]. Moreover, MCMV ie3 was reported to arrest cycling cells in the G1 or in the G2 phase [13]. On the other hand, the murine ie2 gene, which is transcribed from the opposite DNA strand and towards the right end of the viral genome, has no homologue in HCMV [14] and is dispensable for viral growth [15]. Transcriptome comparison of knockout mutants for the MCMV ie1 or the ie2 gene suggested that these MCMV genes may fulfil a redundant function in transcriptional regulation of other viral genes [16]. The murine MIEP consists of a bipartite enhancer flanked by the divergent promoters p1/3 and p2 pointing towards ie1/3 and ie2, respectively [17]. While it is long-established that MCMV may infect a wide variety of cells, and express ie genes even in non-murine cell lines [18] the kinetic of ie gene expression at the single-cell level could not be studied, due to a lack of appropriate reagents. The MCMV genes ie1 and ie2 are expressed in lungs of latently infected mice in a random, asynchronous and asymmetric pattern [19]. In follow-up studies the same group has shown that the major immediate early enhancer (MIE) may act as a genetic switch by preferentially enhancing the transcription of ie1 or ie2, but not of both genes at the same time [20]. However, all these studies were performed by PCR based testing of viral mRNA in lungs of latently infected mice, and thus it remained unclear if the MIEP acts as a genetic switch at the single-cell level and during lytic infection. We performed a single-cell analysis of ie1/3 and ie2 expression during lytic infection by means of a recombinant MCMV carrying a bidirectional fluorescence-reporter MIEP. Infection of different cell types showed that ie1 and ie2 are expressed simultaneously in the vast majority of cells. To compare the expression of the immediate early genes in proliferating and non-proliferation cells we generated a new conditionally-immortalized cell line, but did not observe a difference in ie1/3 or ie2 reporter gene expression, although the virus proliferated much more vigorously in non-cycling cells. In contrast, analysis of different cell types revealed that ie2 accumulates predominantly in macrophages, whereas ie1/3 expression is more pronounced in fibroblasts or endothelial cells.

## Results

### Generation and characterization of conditionally immortalized liver sinusoidal endothelial cells

**Figure 1:**
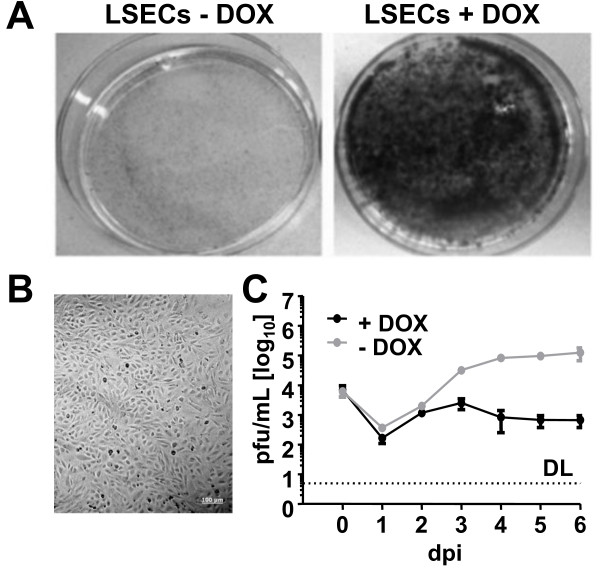
**Growth and susceptibility to MCMV infection of the conditionally immortalized liver sinusoidal endothelial cell line LSEC-uniLT (LSEC). (A)** 10^3^ LSEC-uniLT were cultured for 10 days in 10 cm Petri dishes in the presence of 2 μg/ml doxycycline (+DOX) or in its absence (-DOX). Representative crystal violet stains visualizing cell proliferation are shown. **(B)** Representative brightfield microscopy picture of confluent LSEC-uniLT is shown. **(C)** LSEC-uniLT were infected at 0.1 MOI of MCMV in presence or absence of doxycycline and virus growth was monitored by plaque assay of supernatants at indicated time-points post infection. The average PFU/ml +/- SD of three experiments is shown. The limit of detection (DL) is marked by the dashed line.

Liver sinusoidal endothelial cells (LSEC) are a site of MCMV latency [21], and hence highly relevant for its biology. To our knowledge, an MCMV-permissive LSEC line is not available. Transduction of doxycycline-controlled expression cassettes encoding immortalizing genes such as SV40 T antigen can conditionally immortalize murine cells [22,23]. Cell lines established with this protocol express T antigen in a doxycycline-dependent fashion, allowing the control of the cell cycle and proliferation. Thus, this strategy was applied to immortalize murine LSECs. CD146 positive endothelial cells were isolated from mouse liver and infected with lentiviral vectors encoding doxycycline-dependent expression units of SV40 T antigen (see Methods for details). Thereupon, the cells were grown and maintained in the presence of doxycycline, resulting in a pool of fast growing cells. The proliferative activity of these cells was assessed upon cultivation in presence and absence of doxycycline. In the cell line LSEC-uniLT, proliferation was completely abrogated in the absence of doxycycline for at least 10 days while the presence of doxycycline allowed rapid cell proliferation (Figure 1A). The effect of doxycycline on LSEC-uniLT proliferation was confirmed by antibody staining for Ki-67, a marker of cell cycling that is rapidly down regulated when cells enter the G_0_ phase of the cell cycle [24]. Flow cytometric analysis revealed the presence of Ki-67 only in the presence of doxycycline whereas it was undetectable upon doxycycline retraction (Additional file 1: Figure S1A). Importantly, non-cycling LSECs cultivated in the absence of doxycycline were not senescent, as evidenced by β-Gal staining (Additional file 1: Figure S1C) and doxycycline reintroduction to culture medium re-started Ki-67 expression in LSECs (Additional file 1: Figure S1B) and their replication (data not shown). Thus, the growth of the immortalized cells could be strictly controlled by doxycycline supply or retraction, without resulting in cellular senescence.

LSEC-uniLT cells showed several phenotype and functional characteristics of liver sinusoidal endothelial cells. Confluent cells formed a polygonal flat “cobblestone” monolayer - characteristic of cultured ECs (Figure 1B). Furthermore LSEC-uniLTs expressed the endothelial cell surface markers CD105 and CD146 (Additional file 1: Figure S1D) and could take up high amounts of low-density lipoproteins (Additional file 1: Figure S1E), a function typical of LSEC, but not other endothelial cells [25].

The cells were able to grow while anchored to gelatine, but not in a soft agar matrix, and clusters of cells were not observed upon passaging (data not shown), strongly arguing that LSEC-uniLT were not transformed.

Finally, LSEC-uniLT were tested for permissiveness to MCMV infection. Cells were infected in the presence or absence of doxycycline with a low multiplicity of infection (0.1 MOI) with wild-type MCMV (MCMV-wt) and infectious virus titer in the supernatants was assessed by plaque assay on days 0 to 6 post infection. MCMV replication was markedly enhanced in non-cycling LSEC-uniLT, arguing that viral growth and/or spread may be compromised in cycling LSEC-uniLT (Figure 1B).

### Generation and characterization of a reporter virus expressing YFP and tdTomato under the control of the bidirectional major immediate early promoter MIEP

**Figure 2:**
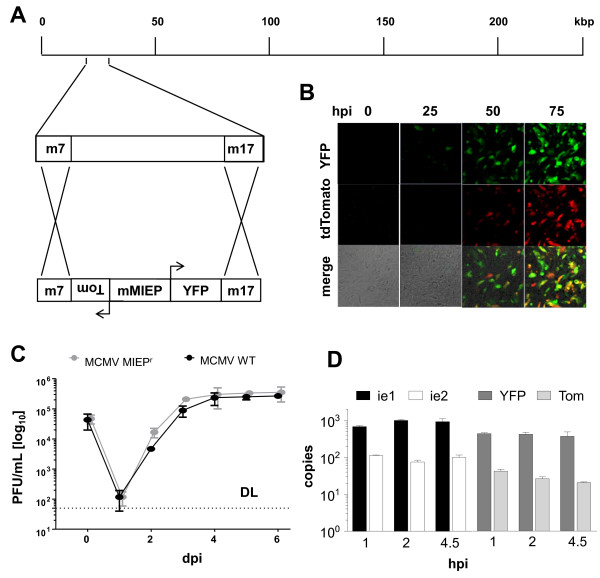
**Generation and characterization of the reporter MCMV MIEP**^**r**^**. (A)** Graphic representation of the MIEP^r^ reporter construct and its integration into the MCMV genome. The bidirectional major immediate early promoter enhancer (MIEP) was flanked by the yellow fluorescent protein (YFP) and tdTomato (Tom) and inserted ectopically into the MCMV genome, replacing the viral genomic region between the genes m07 and m17. **(B)** LSEC-uniLT were infected with 1 MOI of MIEP^r^ and representative pictures were visualized by epifluorescence microscopy at indicated hours post infection (hpi) are shown. **(C)** NIH-3 T3 cells were infected with MIEP^r^ or MCMV wild type (WT) at an MOI of 0.1 and virus growth was monitored by plaque assay of cell supernatants at indicated days post infection (dpi). Averages (+/- SD) from three independent experiments are shown. The dashed line represents the limit of detection (DL). **(D)** Endogenous (ie1 and ie2) and reporter (YFP and Tom) transcripts were measured by qRT-PCR. The cDNA was synthesized from RNA obtained from LSEC-uniLT infected at an MOI of 10 for the indicated hours post infection (hpi). Copy numbers were normalized to GAPDH and are represented as averages (+/- SD) from three independent experiments.

We generated a reporter construct containing the entire MCMV MIEP region, from the start codon of the ie1/3 transcriptional unit to the start codon of the ie2 gene. Thereupon, we fused this construct to reporter genes, a yellow fluorescent protein (YFP) expressed in the ie1/3 orientation and a tdTomato fused to the ie2 start codon (Figure 2A). The entire construct was inserted ectopically into the MCMV genome by site-directed recombination of viral genomes maintained in *E. coli* as bacterial artificial chromosomes (BAC). Since the construct replaced the viral genes m7 to m17, the size of the recombinant viral genome was in essence identical to the parental MCMV strain (Figure 2A). The newly generated MIEP reporter virus was named MCMV-MIEP^r^ and most infected cells appeared to express YFP and tdTomato simultaneously, although some expressed YFP or tdTomato alone (Figure 2B). We tested MCMV-MIEP^r^ for growth on NIH-3 T3 cells by infecting them at an MOI of 0.1 of MCMV-MIEP^r^ or MCMV-wt. Supernatants were tested for infectious virus at days 0-6 post infection by plaque assay on MEF cells. Results indicated that *in vitro* replication of the MIEP^r^ is not attenuated in comparison to MCMV-wt (Figure 2C).

While our construct contained the entire MCMV MIEP, it was formally possible that some regulatory elements driving ie1/3 or ie3 expression are located outside of this region. Hence, to test if the expression of the reporter genes matches the expression of the endogenous MCMV genes ie1 and ie2, we compared the RNA copy number of the reporter genes and their endogenous pendants. We isolated RNA from infected LSEC-uniLT (see Methods) at 1, 2, and 4.5 hours post infection (hpi). The RNA was reversely transcribed and used in quantitative real time PCR. The copy numbers of YFP, tdTomato, ie1, and ie2 were normalized to the cellular gene GAPDH. Both reporter genes were expressed at slightly lower copy numbers than the viral genes under the same promoter, but both genes could be detected at the earliest times post infection and the ratio between YFP and tdTomato mRNA expression matched closely the ie1 to ie2 ratio at all time-points tested (Figure 2D). Hence, we concluded that our reporter system recapitulates the essential features of the MIEP expression.

**Figure 3:**
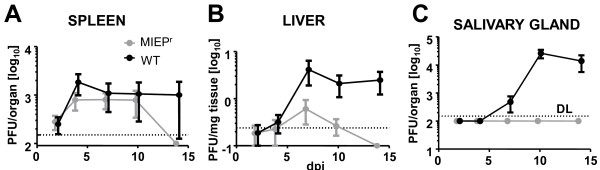
***In vivo *growth of MCMV MIEP**^**r**^**.** Growth of MCMV WT (black line) and MIEP^r^ (grey line) in organ homogenates of **(A)** spleen, **(B)** liver and **(C)** salivary glands of C57BL/6 mice on day 2, 4, 7, 10 and 14 post infection. Each data point depicts the mean obtained from 5 mice and error bars indicate SEM.

While MIEP^r^ MCMV growth was not attenuated *in vitro*, there was a discrete growth defect upon systemic *in vivo* infection (Figure 3). C57BL/6 mice were intraperitoneally infected with MIEP^r^ MCMV and compared to MCMV-wt infected littermates. Similar viral loads could be observed in the spleen during the first ten days, but MIEP^r^ was cleared by day 14, while MCMV-wt could still be detected at this time point (Figure 3A). MIEP^r^ was significantly attenuated and rapidly clear from the liver, but it was still detectable until day 7 post infection, arguing for a relative, but not absolute loss of fitness (Figure 3B). The attenuation was most prominent in the salivary glands, where no infectious MIEP^r^ was detected at any time point (Figure 3C). Importantly, YFP and tdTomato expression could still be detected in virtually all MIEP^r^ viral plaques obtained by virus titration of infected organs (data not shown); arguing that *in vivo* virus passaging did not result in mutations and loss of expression of the reporter genes.

### Comparison of MCMV-MIEP^r^ infected LSEC-uniLT in cycling and G_0_-arrested cells

**Figure 4:**
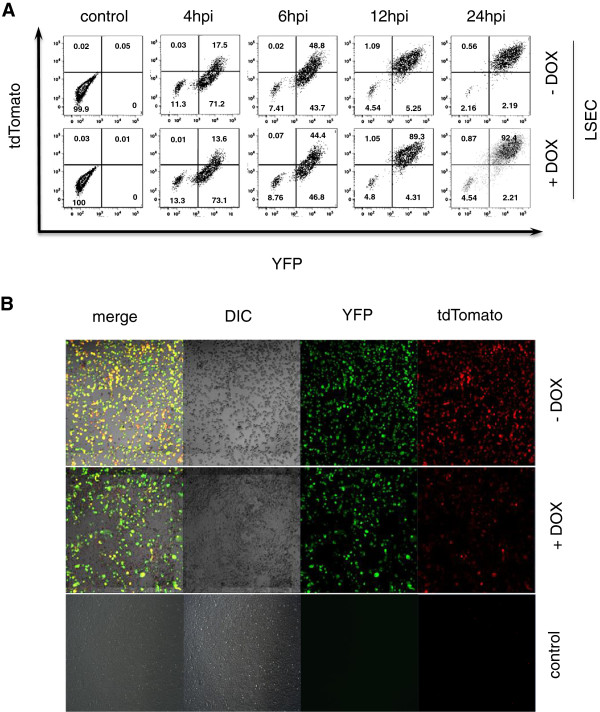
**Cell proliferation has no effect on the early reporter gene expression of the MIEP**^**r**^**. (A)** LSEC-uniLT (LSEC) were cultured in the presence (+ DOX) or absence (- DOX) of doxycycline. Cells were infected with an MOI of 10 and analyzed at 0, 4, 6, 12 and 24 hpi by flow cytometry. Representative dot blots of tdTomato and YFP expression are shown. **(B)** LSEC-uniLT were cultured in the presence (+DOX) or absence (-DOX) of doxycycline and infected with an MOI of 1 or left uninfected (control). At day 5 post infection confocal images were acquired to visualize cells by differential interference contrast (DIC) and the fluorescent reporters (YFP, tdTomato) by fluorescence imaging.

By infecting LSEC at a low MOI with wt MCMV we have observed differences between proliferating and quiescent cells (Figure 1C). To test if cycling cells were less permissive for virus infection, we infected LSEC-uniLT cells in the presence or absence of doxycycline at an MOI of 10 with the MIEP^r^, and compared early time points at 4 h, 6 h, 12 h, and 24 h post infection by flow cytometry (Figure 4A). YFP preceded the tdTomato signal in both the cycling and the non-cycling cells, and both genes were expressed in the majority of cells by 24 hours post infection, but we did not notice a difference in marker gene expression in cycling or non-cycling cells, arguing that cell cycling did not influence the MIEP-driven gene expression at early time points post infection. Similarly, doxycycline had no effect on viral gene expression in NIH-3 T3 cells (data not shown). Since the difference in viral replication was manifested several days post infection (see Figure 1C), we analysed the reporter gene expression and the cytopathic effect at day five post infection (Figure 4B). Proliferating and G_0_-arrested LSEC-uniLT were infected at an MOI of 1 and analysed by fluorescence microscopy and phase contrast microscopy. G_0_ arrested LSEC-uniLT were thoroughly infected by day 5 post infection and virtually all cells expressed the reporter fluorophores and exhibited changes in morphology. On the other hand, approximately two third of the cycling cells showed no signs of infection, neither by cytopathic effect nor by fluorescent gene expression (Figure 4B).

### MCMV-MIEP^r^ gene-expression profiles in different cell types

**Figure 5:**
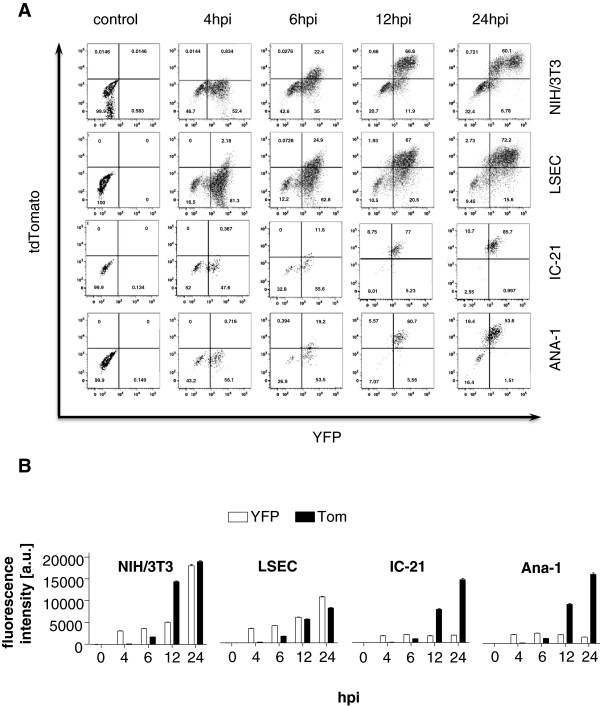
**Comparison of fluorescence profiles in different cell lines infected with the MIEP**^**r**^**.** NIH/3T3, LSEC-uniLT (LSEC), IC-21, and Ana-1 cells were infected with MIEP^r^ at an MOI of 10 and analysed by flow cytometry 0, 4, 6, 12 and 24 hpi. **(A)** Representative dot blots of tdTomato and YFP expression are shown. **(B)** Mean fluorescence intensities of YFP and tdTomato (+ SEM) for the indicated cell lines and times post infection are shown as histograms.

To test if flow cytometric analysis of virus-infected cells always results in identical patterns of ie1/3 and ie2 gene expression, we compared LSEC-uniLT cells to other cell types that are permissive for MCMV infection. We infected NIH-3 T3 fibroblasts and two macrophage cell lines in use in our lab [26] with MIEP^r^ and analyzed the YFP and tdTomato expression until 24 hpi (Figure 5A,B). All cell lines expressed the YFP reporter protein already at 4 hpi, followed by tdTomato expression at 6 hpi or later. The tdTomato mean fluorescence intensity (MFI) increased progressively for the duration of the experiment in all cell types. In contrast to macrophages, the MFI of the YFP signal continued to increase in LSEC-uniLT and fibroblasts until 24 hpi. Interestingly, the intensity of the YFP signal at 24 hpi was the strongest in NIH-3 T3 cells, followed by the LSEC-uniLT. On the other hand, in macrophages the tdTomato MFI increased by 12 hpi to values that were several folds higher than the MFI of the YFP signal, and the tdTomato/YFP signal ratio increased even more by 24 hpi. In conclusion, our data indicated that the gene expression pattern of MIEP driven genes differs between various cell types, and that the expression controlled by the MCMV ie2 promoter is more pronounced in macrophages than in fibroblasts or endothelial cells, but also that cell cycling did not affect the ratio of tdTomato/YFP signals.

### Single cell analysis of MIEP^r^ infected LSEC

**Figure 6:**
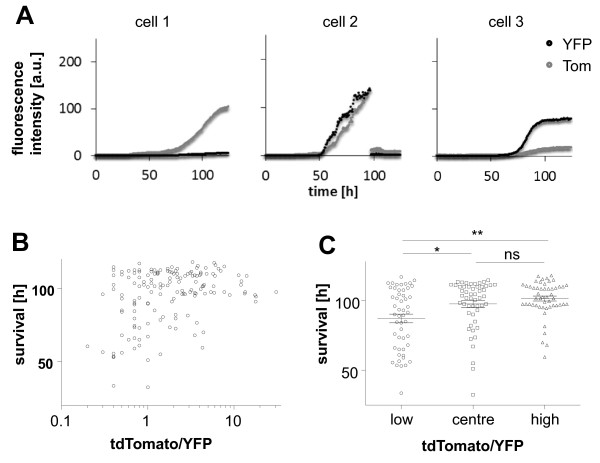
**Live cell imaging and single cell analysis of LSEC-uniLT infected with the MIEP**^**r**^**.** Cells were infected at an MOI of 10 and monitored by fluorescence microscopy in a CO_2_ regulated chamber for 5 days. **(A)** Representative time courses of fluorescence intensities for YFP and tdTomato (Tom) in individual LSEC-uniLT. The signal intensity is represented on the ordinate, whereas the time post infection is shown on the abscissa. **(B)** The median fluorescence intensity ratio of tdTomato/YFP was established for individual infected cells and plotted against the survival time of the infected cell. **(C)** LSEC-uniLT shown in panel B were subdivided into terciles according to their median tdTomato/YFP-ratio. Symbols show survival times of individual cells in the lower (1), centre (2) and upper tercile (3), while horizontal lines show group average (+/- SEM) values. * and ** denote p values below 0.05 and 0.0035, respectively, according to Kruskal-Wallis followed by Dunns post-analysis test.

LSEC-uniLT populations infected with MCMV-MIEP^r^ showed a wide range of fluorescence intensities for either reporter, as observed by microscopy (Figure 2B) or flow cytometry (Figures 4 and 5). To investigate the kinetic of this phenomenon in individual cells, we monitored single LSEC-uniLT cells by time-lapse microscopy for up to five days following infection with MIEP^r^. While most cells expressed both fluorophores, consistent with the kinetics described in Figures 4 and 5, some cells showed polarized expression of tdTomato or YFP (Figure 6A). It is important to note that monitoring of fluorescence expression by time-lapse microscopy allowed only a 2.5 log dynamic range of detection, while flow cytometry allowed us to discriminate very discrete fluorescence signals in the low range, but could not be used to monitor the same cells in a kinetic fashion. Interestingly, the majority of infected cells lived longer than 96 h, and cell death could be visualised by the rapid loss of fluorescent signal (please note the middle panel of Figure 6A as an example). We correlated the tdTomato/YFP ratios with survival time and noticed that infected cells with a shorter survival time tended to be cells with a low ratio of tdTomato/YFP expression (Figure 6B), and Spearman test resulted in a highly significant correlation index (r = 0.28, p = 0.0005), arguing that LSEC-uniLT that expressed relatively more YFP than tdTomato lysed earlier than cells that expressed more tdTomato. Clustering the cells according to their tdTomato/YFP ratio in terciles with low, medium and high ratios allowed us to compare their survival time. We observed that survival of infected cells was significantly shorter in cells from the lower tercile than in cells from the middle or high tercile, but we saw no differences between cells in the mid and in the high tercile (Figure 6C). Therefore, our data indicated that cells with a higher p1/3 activity might be lysed faster than the cells expressing more abundantly the ie2 gene.

## Discussion

In differentiated tissue, most cells rest in the G_0_ phase of the cell cycle. Hence, it has been assumed that CMV infects resting cells *in vivo*, which is in accordance with evidence that transformed cells are less permissive for CMV replication than primary ones [18]. While the pattern of immediate-early gene expression in cycling cell lines may differ from the *in vivo* situation [27], the effect of cell cycling on viral gene expression was never tested in detail. The effects of cell cycle on viral gene expression *in vitro* have been studied by synchronization and arrest of the cell cycle in the G_0_ phase by serum starvation prior to CMV infection [27,28], but this method results in increased cell death when applied over several days, and hence it is unsuitable to monitor viral replication in resting cells over an extended period of time. On the other hand, it was reported that the MCMV ie3 gene expression causes an arrest of synchronized cycling cells in the G_0_/G_1_- and G_2_-phase [13], implying that any cycling cell becomes a resting one, upon MCMV infection. It remained unclear if cycling may affect immediate-early gene expression prior to ie3 gene expression and if resting non-starved cells may allow better viral replication. We have generated a conditionally immortalized liver sinusoidal endothelial cell line (LSEC-uniLT) that proliferates only in the presence of doxycycline and can be cultured up to 21 days in the G_0_ phase upon its retraction, and observed a 100 fold increase of viral particles released from cells arrested in the G_0_-phase over the cycling cell line. While we cannot formally exclude that doxycycline inhibited MCMV growth in our cell line, others have shown that doxycycline addition does not impair MCMV replication in cultured cells [29]. Therefore, our data support the idea that MCMV replication is impaired in cycling cells.

MIEP^r^ reporter genes were expressed five days post infection in all non-cycling LSEC whereas the majority of cycling LSEC showed no sign of infection (Figure 4B). Since the reporter genes showed identical expression pattern within the first 24 h of infection in proliferating and in the G_0_-phase arrested LSEC-uniLT (Figure 4A), we assume that poor replication in cycling cells was not due to defects in cell entry or inhibition of MIEP expression. It remains unclear if the virus replicative cycle was blocked in cycling cells at the early or late stage, or if the virions that came out of cycling cells were less infective.

Various reporter systems have been used in the past to monitor cell tropism of MCMV infection. GFP expression under the control of the human CMV-promoter [30,31] allowed the tracking of cell types that can be infected by different routes of infection or of cells which may help to disseminate the virus [32]. Marquardt et al. used a more sophisticated reporter system by coupling mCherry and the Gaussia luciferase via a picornavirus P2A cleavage site under control of the murine MIEP to study single cell reactivation from latency in latent lung tissue [33]. However, these reporter systems used a truncated MIEP to drive gene expression, which expressed the genes in one direction only and could not reflect accurately the ie1 gene expression, because most of the enhancer was missing. Others have studied the localization and protein-protein interaction studies of IE1 and IE3 by GFP fusion to these genes [34-36], but these systems could not allow the comparison of ie1 and ie2 gene expression patterns and could not exclude that the fusion resulted in alterations in the biology of the targeted proteins. We generated a fluorescent reporter virus that expresses YFP and tdTomato instead of ie1/3 and ie2, and that is regulated by the full-length murine MIEP inserted ectopically as an additional copy, to circumvent alterations or loss of function of ie1/3 or ie2. The *in vivo* replication of MIEP^r^ MCMV was partially impaired, but the mutant virus was nevertheless able to productively replicate in the spleen and liver of infected mice. Therefore, the use of MIEP^r^ MCMV in animal experiments will necessarily be restricted to experimental systems that do not depend on maintained replicative fitness. It is tempting to speculate that this virus might be used to study viral latency, since its ability to infect quiescent LSECs was entirely maintained. On the other hand, the *in vitro* growth was indistinguishable from the parental MCMV clone, arguing that MIEP^r^ MCMV might be used with fewer restrictions in tissue cultures systems. So far, differences in the expression of genes driven by the MIEP were studied by means of antibody staining of brain sections against IE1, IE2, and IE3 in neonatal mice infected with MCMV [37] and by PCR analysis of gene expression in latently infected lungs [17,20,27]. To the best of our knowledge, this is the first analysis of ie1/3 and ie2 expression kinetics in single cells and the first comparison of fibroblast expression pattern to those in LSEC or macrophage cell lines. Since MCMV replicates to higher titers in fibroblasts and endothelial cells than in macrophages, it is tempting to speculate that differences in ie2 expression levels contribute to a difference in permissiveness of cells for productive MCMV infection, yet this hypothesis would need validation in further studies.

We generated an LSEC cell line that enabled us to study cell cycle dependent infection, showing that cell cycling does not affect MIEP gene expression, although it severely limits virus growth. We used for this purpose an LSEC line because endothelial cells are a physiological site of CMV infection [38], and LSECs were shown to be the major site of MCMV latency in the liver, pointing out the importance of this particular cell type during natural CMV infection [21]. The fact that LSECs are permissive for lytical MCMV replication *in vitro* but are sites of latency *in vivo* might indicate that MCMV latency in LSECs depends on the immune system and its interaction with the infected cells. Further studies are necessary to clarify this intriguing possibility. By monitoring the infection of single LSEC-uniLT by time-lapse fluorescence microscopy we observed that the fluorescent signals preceded the detachment and rounding of cells by several hours. Moreover, we noticed, that cells followed divergent temporal patterns of reporter expression. While the majority of the cells expressed both fluorophores at high levels, some expressed predominantly one or the other. It has been proposed, based on quantitative RT-PCR in lungs of latently infected mice [17,20,27] that MIEP may acts as a bidirectional switch for ie1 and ie2 expression. In our system we observed a roughly 200-fold variation in p1/3 and p2 promoter activity, reflected by differences in the range of tdTomato/YFP-ratios in microscopy. It is important to note that we could not directly assess which of the two reporter genes was more abundantly expressed, as it was impossible to normalize their fluorescence level directly, but we observed a preponderance of YFP over tdTomato at the mRNA level, which was consistent with the ratio of ie1 and ie2 transcripts.

Correlation of the tdTomato/YFP ratio against the survival time of infected cells showed that cells dying early in the course of the experiment clustered in the fraction with a low tdTomato/YFP ratio. The cause effect relationship of this observation needs clarification in subsequent studies, because it is unclear whether a low ie2/ie1 ratio caused early cell death or whether tdTomato (and consequently ie2) accumulate in long-term infected cells which are not lysed, thus increasing the tdTomato to YFP ratio. While the function of MCMV ie2 remains unknown, it is tempting to speculate that it may protect infected cells from lysis.

## Conclusions

In conclusion, we showed a novel fully viable reporter MCMV, carrying the full-length MCMV MIEP, and therefore all of the binding sites for various cellular transcription factors that regulate its activity during lytic and latent infection. The transcription kinetic of reporter genes matched the transcription of endogenous MCMV genes and this system allowed us to observe that cycling and quiescent endothelial cells showed identical MIEP expression patterns, although MCMV virus grew ~100 fold less well in cycling cells than in the G_0_ arrested ones. On the other hand, we observed a more dominant expression of the p2-driven gene in macrophages than in fibroblasts and endothelial cells and a correlation of early cell lysis with stronger p1/3 over p2 expression. Therefore, our reporter MCMV may be a promising tool to study the factors promoting or suppressing the MIEP expression both during lytic and latent infection.

## Methods

### Ex vivo isolation and conditional immortalization of LSEC lines

Conditionally immortalized liver sinusoidal endothelial cells (LSECs) were generated from RosaConL BALB/c mice [39]. Initial isolation of mouse liver non parenchymal cell (NPC) was performed according to a published protocol [40]. In brief, the liver was perfused with 10 ml liver perfusion medium (Gibco-Invitrogen, Paisley, UK) and with 5 ml liver digestion medium (Gibco-Invitrogen, Paisley, UK). Upon removal of the liver from the mouse, the liver was cut in small pieces, incubated for 30 min in liver digestion medium and gently pressed through a Nylon 100 μm cell strainer (BD Falcon). Non parenchymal cells (NPC) were separated from parenchymal hepatocytes by centrifugation at 50 × *g* for 5 min. NPC were collected, washed in PBS, resuspended in 40% Percoll (Biochrom), gently overlaid onto 70% Percoll, and centrifuged at 750 × *g* for 20 min. NPC collected from the interface were washed twice and resuspended in PBS/1%FCS.

Upon red blood cell lysis, LSECs were isolated from NPCs by immunomagnetic sorting. For this purpose, 2 × 10^7^ nucleated cells were resuspended in 200 μl PBS/1% FCS along with 20 μl of antimouse-CD146–conjugated magnetic beads (Miltenyi Biotec), incubated for 20 minutes at 4°C and magnetically separated according to the manufacturer’s protocol.

Isolated LSECs were maintained in RPMI supplemented with 10% fetal bovine serum (FBS) (PAN Biotech, Aidenbach Germany), 2.5% HEPES (pH 7.1), penicillin (100 U/mL), streptomycin (100 μg/mL), L-glutamine (2 mM), 1 mM sodium pyruvate and 0.2 mM 2-mercaptoethanol (Gibco-Invitrogen, Paisley, UK) on plates coated with 0.5% gelatin (Sigma, St. Louis, MO, USA). Cells were seeded on flasks or plates and cultivated in an incubator at 37°C, 7% CO_2_ and 5% O_2_, at maximal humidity.

Conditional immortalization of cells was performed based on a protocol previously published for human endothelial cells [41]. For this purpose, lentiviral vector uni-Tag (encoding SV40 T antigen in a doxycycline (Dox) dependent, autoregulated cassette was used to infect LSECs at a multiplicity of infection (MOI) of 1 in the presence of 4 μg/ml of polybrene (Sigma). To increase efficiency, infection was repeated. 24 hours after the second infection, the viral supernatant was removed and Dox was added to the medium to activate the immortalization cassette.

A conditionally immortalized pool of cells was obtained upon cultivation of cells and selection for growth advantage in Dox presence. This cell line was named LSEC-uniLT.

### Generation of the MIEP reporter virus

MCMV^r^ was generated by two-step E/T BAC mutagenesis using an FRT-flanked Kanamycin resistance gene (Kan^R^) essentially as described in [42] with some modifications. Homologous regions derived from the MCMV genes m7 (nucleotide position 6759-6782) and m17 (nucleotide position 15882-15907) were amplified by PCR using the primers KpnISapI-m7 Fwd / AvrII-m7 Rev and EcoRI -m17 Fwd / BglIISapI-m17 Rev (for primer sequences see Additional file 2: Table S1) and inserted into EcoRI and BglII (for m17) or KpnI and AvrII (for m7) restriction sites of the pLitmus28 (New England Biolabs) plasmid, giving rise to pLITMUS-m7 + 17. The tdTomato gene was PCR amplified with primers BamHI-Tom-antis Fwd (containing 57 nucleotides upstream of the MCMV IE2 ATG) and AvrII-Tom-antis Rev (bearing the HSV-1 poly(A) sequence) and cloned into pGEM-T-Easy, resulting in pGEM-Tom. Similarly, the YFP sequence was inserted into pGEM-T-Easy by using the primers EcoRV-MIE-YFP Fwd and EcoRI- YFP Rev that contained 67-bp upstream of the IE1/3 ATG) and the poly(A) of HSV 1, respectively, which resulted in pGEM-YFP. The full-length MCMV Major Immediate Early Enhancer (MIE), the promoter regions and the 5′untranslated sequences of the of the ie1/3 and the ie2 genes, were PCR amplified with primers BamHI-MIE Fwd and EcoRV-MIE Rev and cloned into pGEM-T-Easy, thus resulting in pGEM-MIEP. The KpnI/SacI fragment was excised from the pGEM-YFP and cloned into the same restriction sites in pLitmus-m7 + m17, resulting into pLitmus-YFP. By using the restriction enzymes BamHI and XbaI, the tdTomato sequence was excised from pGEM-Tom and inserted into pLitmus-YFP and the resulting plasmid was named pLitmus-YFP/Tom. The MIEP sequence was too large for a single step insertion and was thus inserted between the YFP and the TdTomato sequence of pLitmus-YFP/Tom in a two-step procedure: First, a 1400 kb fragment was amplified from the pGEM-MIEP using the primers EcoRV-MIE Rev and BamHI-MIE-PacI, upon which the PCR product was digested with EcoRV and BamHI and inserted into the same restriction sites of pLitmus-YFP/Tom . The second 2800 kb fragment was excised from pGEM-MIEP by PacI and BamHI digestion and cloned into the pLitmus construct using the same restriction sites, resulting in pLitmus-YFP-MIEP-Tom. It is important to note that the MIEP sequence was inserted in antisense orientation relative to the m7 and m17 sequences, in order to avoid recombination events in the viral genome. The FRT-flanked Kan cassette was excised from the plasmid pGP704 with EcoRI and inserted between the YFP and m17. The final construct was flanked by the homology parts of m7 and m17, contained the entire MIE from MCMV driving the expression of the fluorescent proteins YFP and tdTomato and a Kan cassette as a selection marker. The construct was linearized by SapI digestion and transformed into E.coli SW102 carrying the MCMV-BAC clone pSM3fr-MCK-2 fl [43]. Homologous recombination was performed by temperature induction of recombinases and clones were selected on Kanamycin (Kan) plates. To eliminate the Kan cassette, *E.coli* that contained the recombinant BAC were transiently transformed with the pcp20 plasmid that expresses a temperature sensitive Flp-recombinase [44] and recombinant clones were selected for Chloramphenicol but not Kanamycin resistance. The sequence of the final construct was validated by sequencing prior to transfection of MEF cells and infectious virus reconstitution.

### Cell lines and virus strains

NIH-3 T3 (ATCC no. CRL-1658), IC-21 (ATCC no. TIB-186), Ana-1 and M2-10B4 (ATCC no. CRL- 1972) were cultured in DMEM supplemented with 10% FBS (Gibco), penicillin (100 U/mL), streptomycin (100 μg/mL), L-glutamine (2 mM) at 37°C, 5% CO_2_, 95% humidity. LSEC-uniLT were maintained in RPMI supplemented with 10% fetal bovine serum (FBS) (PAN Biotech, Aidenbach Germany), 2.5% HEPES (pH 7.1), penicillin (100 U/mL), streptomycin (100 μg/mL), L-glutamine (2 mM), 1 mM sodium pyruvate and 0.2 mM 2-mercaptoethanol (Gibco-Invitrogen, Paisley, UK) on plates coated with 0.1% gelatin (Sigma, St. Louis, MO, USA). Cells were seeded on flasks or plates and cultivated in an incubator at 37°C, 7% CO_2_ and 5% O_2_, at maximal humidity.

MCMV wt (molecular clone MW97.01) [45] and MIEP^r^ were propagated on M2-10B4-cells as described elsewhere [46].

### Ki-67 staining

LSEC-uniLT were cultivated in the presence or absence of doxycycline for three days and subsequently stained with Ki-67-FITC or with isotype control (BD Bioscience). Cells were fixed with IC buffer (eBioscience) and permeabilzed with Perm/Wash buffer (BD Bioscience). The antibody was diluted as recommended by the distributors and the cells were acquired with an Accuri C6 flow cytometer (BD Bioscience). Data analysis was performed with FlowJo software.

### AcLDL uptake

LSEC-uniLT were cultured one day in the absence of doxycycline and were subsequently incubated with 10 μg/mL AcLDL-Alexa488 (Invitrogen) for 4 h. Cells were trypsinized and stained with CD146-PerCpCy5.5 (eBioscience) for 20 min. Cells were washed and acquired in an Accuri C6 cytometer. Data were analyzed with FlowJo software.

### *In vitro* infection of cells and plaque assay

Viral growth was assessed by infecting NIH-3 T3 cells with MCMV MIEP^r^ or MCMV WT at an MOI of 0.1. The virus was removed after 1 h, cells were washed with PBS, supplied with fresh medium and incubated until supernatant harvest, at 0-6 days post infection. Supernatant were stored at -70°C until the titration on MEFs as described previously [47].

For infection with high multiplicity of infection, cells were seeded into multiwell plates or IBIDI μ-Slide 8 well dishes (ibidi labware) one day before infection. At the day of infection, a reference well was trypsinized and cells counted. Virus was thawed on ice, diluted in cell culture medium and added to the cells. Cells were centrifuged for 5 min at 2800xg at ambient temperature, which enhances infection by a factor of 12 (data not shown) [48]. Non-bound virus was removed from the cells and fresh medium was added (for flow-cytometric assays) or medium containing 0.75% (w/v) Methylcellulose (for microscopic analysis) was layered on top.

### *In vivo* infection and quantification of infectious virus in organs

All animal experiments were performed in compliance with the German animal protection law (TierSchG BGBI S. 1105; 25.05.1998). The mice were housed and handled in accordance with good animal practice as defined by FELASA and the national animal welfare body GV-SOLAS. All animal experiments were approved by the responsible state office (Lower Saxony State Office of Consumer Protection and Food Safety) under permit number 33.9-42502-04-11/0426. 6 to 10 weeks old C57BL/6 mice were intraperitoneally infected with 5×10^5^ PFU of tissue culture-derived virus and housed in SPF conditions throughout the experiment. Organs were collected under sterile conditions at indicated time points post infection and stored at -70°C until titration. MCMV from organ homogenates or tissue culture supernatants were titrated on MEFs as described above.

### Senescence staining

LSEC-uniLT were seeded onto 6 well plates and incubated for 7 days in the absence of doxycycline (Dox) under low oxygen conditions. Senescence associated ß-galactodiase activity was measured by a senescence staining kit (InSCREENeX, Germany). In brief, the cells were washed twice with PBS and incubated with Fixation Buffer for 2 min. After two additional washing steps with PBS, the staining solution was added and the cells were incubated for 24 h at 37°C. The human foreskin endothelial cell line FS4LTM [49] was stained as a positive control.

### Flow cytometric analysis

Infected cells were trypsinized at indicated times post infection, and an equal amount of medium containing Hoechst 33258 was added to the samples stored on ice until acquisition (up to one hour). Flow cytometric analysis was performed at an LSR-Fortessa or LSR2, both equipped with lasers emitting monochromatic light at 360 nm to excite Hoechst 33258, 488 nm to excite YFP and 561 nm to excite tdTomato. The fluorescence signals of Hoechst 33258, YFP and tdTomato were detected by selective emission filters 450/50, 525/50, and 582/15, respectively. Data analysis was performed with FlowJo 9.4 (TreeStar).

### Confocal microscopy and life cell imaging

Life cell imaging was performed with a Zeiss LSM 510 Meta confocal microscope equipped with a 365 nm, a 488 nm, and a 564 nm laser to excite Hoechst 33258, YFP and tdTomato. Data analysis was performed by Zen analysis software package (Carl Zeiss, Germany).

### Quantative real-time PCR

RNA was extracted by TRIzol (Invitrogen) according to the manufacturer’s protocol, dissolved in DEPC-treated Water and stored at -80°. For cDNA synthesis 5 μg RNA were first treated with DNAse to remove residual DNA contaminants. Following, DNAse was heat-inactivated at 65°C for 15 min and reverse transcribed using SuperScript II (Invitrogen) with random hexamers and oligoDT primers according to the manufacturer’s instructions. For quantitative Real-Time-PCR, up to 2 μl cDNA were used in a 10 μl reaction using a peqGold Real-Time Mix (PEQlab) with syber-green. PCR was performed in a Roche LightCycler 480 (Roche).

### Statistical analysis

Statistical analysis was performed with Prism 5.04 for Windows, GraphPad Software, San Diego California USA, http://www.graphpad.com.

## Competing interests

The authors declare that they have no competing interests.

## Authors’ contributions

LCS conceived the study. LCS, AW and FD designed the experiments. LCS, DW, AW and FD wrote the manuscript. DW, MB and TM generated the LSEC-uniLT. BA provided the wild-type BAC construct for mutagenesis. FD, AW, IC, JH and MB performed the experiments. All authors read and approved the final manuscript.

## Supplementary Material

Additional file 1: Figure S1 Characterization of LSEC-uniLT. (**A**) Histogram of LSEC-uniLT cultured in the presence (black line) or absence of doxycycline (black fill) and stained with the proliferation marker Ki-67 or with an isotype control (grey fill). (**B**) Histogram of LSEC-uniLT cultured for 3 days in absence and then for 3 more days in the presence of doxycycline. Cells were stained with the Ki-67 (black line) or with the isotype control (grey fill). (**C**) Microscopic pictures of senescent FS4LTM and viable LSEC-uniLT cultured in the absence of doxycycline for 10 days. Senescence was visualized by positive staining for β-Gal. (**D**) Histograms of LSEC-uniLT stained for the cell surface markers CD105 and CD146 (black line) and isotype controls (grey fill). (**E**) AcLDL uptake of LSEC-uniLT. Histogram of LSEC-uniLT cultured in the presence (black line) or absence of AcLDL (grey fill). Click here for file

Additional file 2: Table S1 List of primers used in this study. Click here for file
