# Supplementary figures and images for: A new reporter mouse cytomegalovirus reveals maintained immediate-early gene expression but poor virus replication in cycling liver sinusoidal endothelial cells
Source: Virol J. 2013 Jun 17;10:197. doi: 10.1186/1743-422X-10-197 (PMC3765632; doi:10.1186/1743-422X-10-197)

## Slide 1
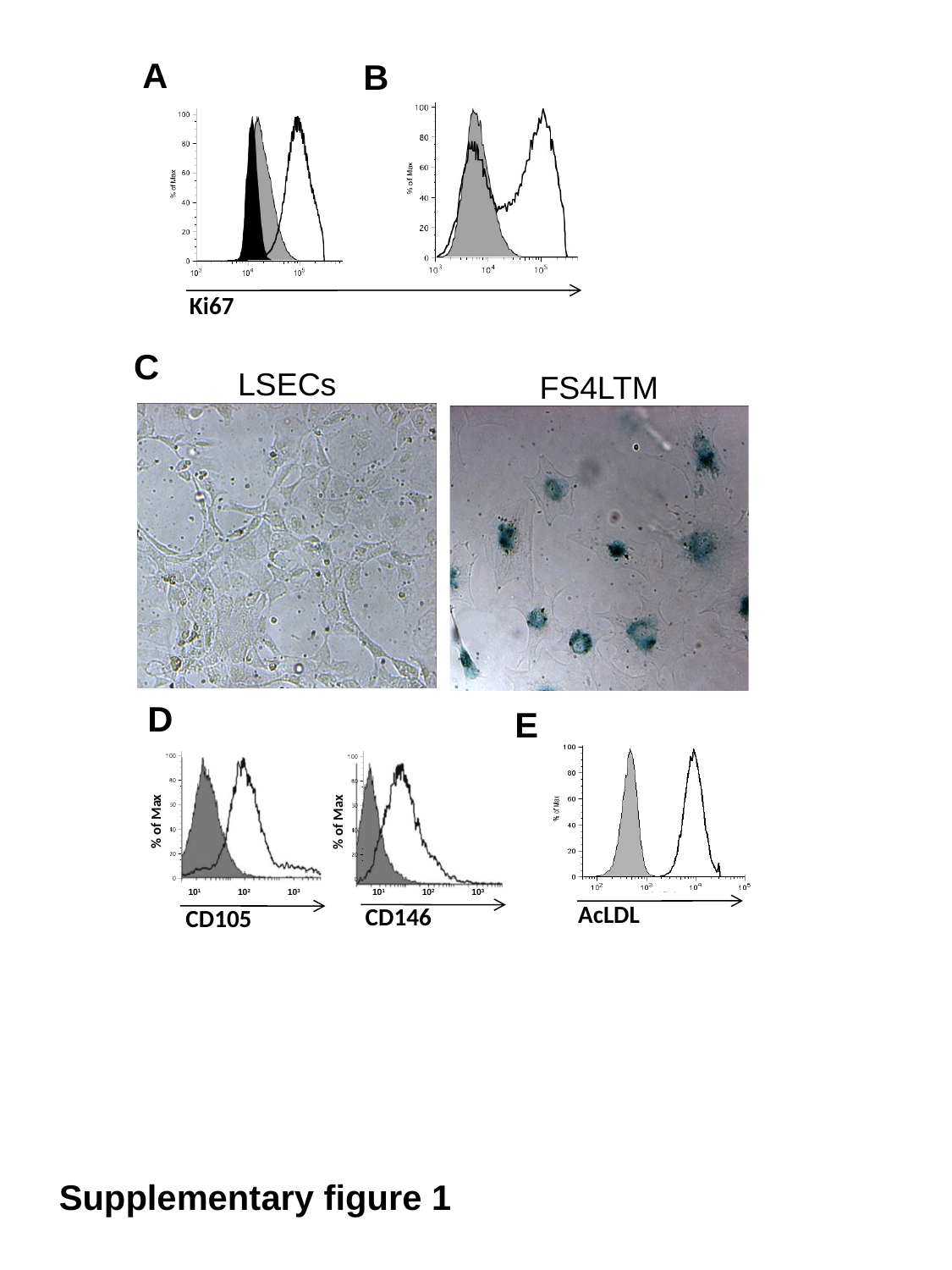

A
B
Ki67
C
LSECs
FS4LTM
D
E
% of Max
101
102
103
CD105
% of Max
101
102
103
CD146
AcLDL
Supplementary figure 1

Supplement: Additional file 1: Figure S1 — Characterization of LSEC-uniLT. (A) Histogram of LSEC-uniLT cultured in the presence (black line) or absence of doxycycline (black fill) and stained with the proliferation marker Ki-67 or with an isotype control (grey fill). (B) Histogram of LSEC-uniLT cultured for 3 days in absence and then for 3 more days in the presence of doxycycline. Cells were stained with the Ki-67 (black line) or with the isotype control (grey fill). (C) Microscopic pictures of senescent FS4LTM and viable LSEC-uniLT cultured in the absence of doxycycline for 10 days. Senescence was visualized by positive staining for β-Gal. (D) Histograms of LSEC-uniLT stained for the cell surface markers CD105 and CD146 (black line) and isotype controls (grey fill). (E) AcLDL uptake of LSEC-uniLT. Histogram of LSEC-uniLT cultured in the presence (black line) or absence of AcLDL (grey fill). [file 1743-422X-10-197-S1.ppt]
