# Supplementary material for: A new reporter mouse cytomegalovirus reveals maintained immediate-early gene expression but poor virus replication in cycling liver sinusoidal endothelial cells
Source: Virol J. 2013 Jun 17;10:197. doi: 10.1186/1743-422X-10-197 (PMC3765632; doi:10.1186/1743-422X-10-197)
Supplement: Additional file 2: Table S1 — List of primers used in this study. [file 1743-422X-10-197-S2.docx]

**Table S1: List of primers used in this study**

| **Primer name** | **sequence** |
| --- | --- |
| \| KpnISapIm7Fwd \|  \| \| --- \| --- \| | GGGGTACCGCTCTTCgcctttgtttagcatgactttta |
| AvrII-m7 Rev | GGGGCCTAGGgtggtgatgagttttctgatggt |
| EcoRI -m17 Fwd | GGAATTCatcaaacagatagcaaaaatgggta |
| BglIISapIm17Rev | GAAGATCTGAAGAGCataccccttacggaatgtataccag |
| BamHITomantisFwd | CGGGATCCGGGGGCGTGAGGGACGCGACTGCTCACGGTTCTGTTTGTCTGTAGATatggtgagcaagggcgaggaggtcatcaaag |
| AvrIITomantisRe | GGGGCCTAGGTTTATTttacttgtacagctcgtccatgccgtacag |
| EcoRVMIEyfpFwd | ATCATCTTCTGGTCTCTGTGGACATCTGTTGATGATAAAAAATTATATTTTTTTAGAGAGATGGTGAGCAAGGGCGAGGAGCTGTTCACC |
| EcoRI YFP Rev | GGAATTCTTTATTTTACTTGTACAGCTCGTCCATGCCGAGAGTGATCC |
| BamHI MIE Fwd | CGGGATCCgagagactctcactacagctgctac |
| EcoRV MIE Rev | ATCtaaagtatacagtgctaataatcatcagtg |
| BamHI MIE-PacI | CGGGATCCGTTTTTCCAGCCAATTTAATTAAAACGCC |
| EYFP fwd | AAGTTCATCTGCACCACCG |
| EYFP rev | TCCTTGAAGAAGATGGTGCG |
| tdTomato fwd | GCGCTCGGTGGAGGCCTCCCAGC |
| tdTomato rev | GGAGCGCGTGATGAACTTCGAGGACGG |
| Ie1 fwd | GGCTGATTGATAGTTCTGTTTTATC |
| Ie1 rev | CTCATGGACCGCATCGCTGAC |
| Ie2 fwd | GCGTCAGTCTGAAGAACAAAGG |
| Ie2 rev | GATACGACCCTACCTACGTTAACG |
